# Stemazole Promotes Oligodendrocyte Precursor Cell Survival In Vitro and Remyelination In Vivo

**DOI:** 10.3390/ijms231810756

**Published:** 2022-09-15

**Authors:** Yizi Zhu, Mingzhu Chen, Yubo Zhang, Mei Han

**Affiliations:** Key Laboratory of Radiopharmaceuticals, Ministry of Education, College of Chemistry, Beijing Normal University, Beijing 100875, China

**Keywords:** multiple sclerosis, remyelination, oligodendrocyte precursor cells, stemazole

## Abstract

Maintaining the normal function of oligodendrocyte precursor cells (OPCs) and protecting OPCs from damage is the basis of myelin regeneration in multiple sclerosis (MS). In this paper, we investigated the effect of stemazole, a novel small molecule, on the promotion of oligodendrocyte precursor cell survival and remyelination. The results show that stemazole enhanced the survival rate and the number of clone formation in a dose-dependent manner and decreased the percentage of cell apoptosis. In particular, the number of cell clones was increased up to 6-fold (*p* < 0.001) in the stemazole group compared with the control group. In vivo, we assessed the effect of stemazole on recovering the motor dysfunction and demyelination induced by cuprizone (CPZ). The results show that stemazole promoted the recovery of motor dysfunction and the repair of myelin sheaths. Compared with the CPZ group, the stemazole group showed a 30.46% increase in the myelin area (*p* < 0.001), a 37.08% increase in MBP expression (*p* < 0.01), and a 1.66-fold increase in Olig2 expression (*p* < 0.001). Histologically, stemazole had a better effect than the positive control drugs. In conclusion, stemazole promoted OPC survival in vitro and remyelination in vivo, suggesting that this compound may be used as a therapeutic agent against demyelinating disease.

## 1. Introduction

Multiple sclerosis (MS) is a chronic demyelinating disease of the central nervous system that gives rise to focal lesions in the gray and white matter and diffuse neurodegeneration in the entire brain [1,2]. In 2020, 2.8 million individuals were living with MS worldwide [3]. MS is a lifelong, chronic and recurrent disease of the central nervous system that can lead to paralysis, blindness and even death without timely treatment [4].

The pathology of MS involves the reduction in nerve cells, the destruction of myelin sheaths and the disintegration of axons in the white and gray matter [4]. Remyelination is the process in which new myelin sheaths are restored to demyelinated axons, reinstating saltatory conduction and resolving functional deficits [5]. The formation of myelin originates from the proliferation, migration and differentiation of oligodendrocyte precursor cells (OPCs) [6,7]. The failure of remyelination is related to OPCs that do not function as they should [8,9]. Therefore, maintaining the normal function of cells and protecting them from injury is the basis of myelin regeneration [9,10].

As a complex disease, some hypotheses of MS pathogenesis, including primary neurodegenerative disease and primary inflammatory disease, have been proposed. According to the different pathogenesis hypotheses, treatment strategies of neuroprotection and myelin regeneration therapy and immunotherapy have been proposed [11,12]. Currently, based on immunotherapy, many disease-modifying therapy (DMT) drugs have been developed. Most of these drugs only relieve symptoms and reduce the recurrence of the disease by modulating the immune response and reducing inflammation but fail to address the desired long-term outcome of remyelination and the recovery of neurological function. Due to the limitations of immunotherapy, MS therapy targeting neuroprotection and myelin regeneration has attracted increasing attention. The core of neuroprotection and myelin regeneration therapy is to improve nerve injury and restore myelin regeneration [1]. Dimethyl fumarate (DMF, BG-12) is an approved drug with neuroprotective effects [13,14]. It can reduce the recurrence rate and prolong the progression of disability [14,15]. Thus, DMF was used as a positive control drug to compare the neuroprotective effect of stemazole on multiple sclerosis.

Based on different hypotheses, different animal model studies have focused on experimental autoimmune encephalomyelitis (EAE) and CPZ models. The EAE model is mainly applied to screen drugs related to the immune system [16]. The CPZ model is a toxin-induced model that simulates the process of myelin damage and myelin regeneration [17] which is suitable for screening drugs with neuroprotective and remyelination functions [18]. Based on the PubMed database, we searched for relevant studies of demyelination animal models published in the last 20 years. The results show that the number of studies related to the EAE model tended to level off as of 2013, however, the number of CPZ model studies has shown a sharp increase since 2013. The increasing number of CPZ model studies indicates the increasing importance of neuroprotection and myelin regeneration therapies. In addition, the increasing number suggests that this is a suitable animal model for simulating behavioral and histological myelin injuries.

Stemazole (C_9_H_9_N_5_OS_2_, chemical name: N-(4-(4,5-dihydro-5-thioxo-1,3,4-oxadiazol-2-yl)phenyl) hydrazine carbothioamide) is a novel small molecule that has neuroprotective pharmacological activity [19,20,21,22,23] and promotes cell survival [23,24]. Stemazole can promote the survival of stem cells by preventing apoptosis under nutrient deficiency or injury conditions [23]. Additionally, stemazole can significantly improve memory impairment in a rat model of AD [20] and has a therapeutic effect on motor nerve damage in a PD mouse model [21]. Stemazole has revealed new pharmacological activity in preventing and treating neurodegenerative diseases by promoting the repair potential of stem cells. Due to the pharmacological activity of stemazole, we hypothesized that it may promote myelin regeneration, improve nerve injury and restore nerve function in the treatment of MS [21,22]. In this study, we investigated whether: (i) stemazole could promote OPC survival and (ii) stemazole could sufficiently improve mouse motor dysfunction and remyelination in a CPZ-mediated demyelination animal model.

## 2. Results

### 2.1. Stemazole Promotes Oligodendrocyte Precursor Cell Viability

**Figure 1:**
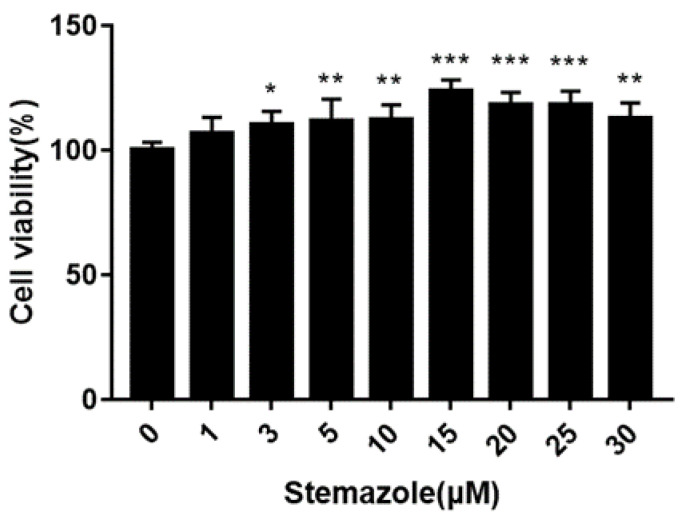
OPCs were treated with stemazole for 4 days, and the effects were analyzed with a Cell Titer-Glo luminescent cell viability assay. Stemazole promotes cell viability in OPCs. Untreated cells were used as the control (100%). Data represent the means ± SD (*n* = 6 duplicates), * *p* < 0.05, ** *p* < 0.01, *** *p* < 0.001. vs. 0 μM group.

To investigate the ability of stemazole to promote cell survival and viability, cells were examined after 4 days by a cell viability assay. To explore whether stemazole could affect OPC survival, we measured the viability of OPCs cultured with different concentrations of stemazole (1–30 μM) (Figure 1). Compared to OPCs cultured without stemazole, the viability of OPCs was significantly enhanced by stemazole: at concentrations of 3, 5, 10, 15, 20, 25, and 30 μM, the cell viability increases were 10.37%, 11.63%, 12.37%, 23.78%, 18%, 18.03%, and 12.56%, respectively. The results indicate that stemazole can increase the viability of OPCs in a dose-dependent manner.

### 2.2. Stemazole Enhances the Survival of Disaggregated Oligodendrocyte Precursor Cells

**Figure 2:**
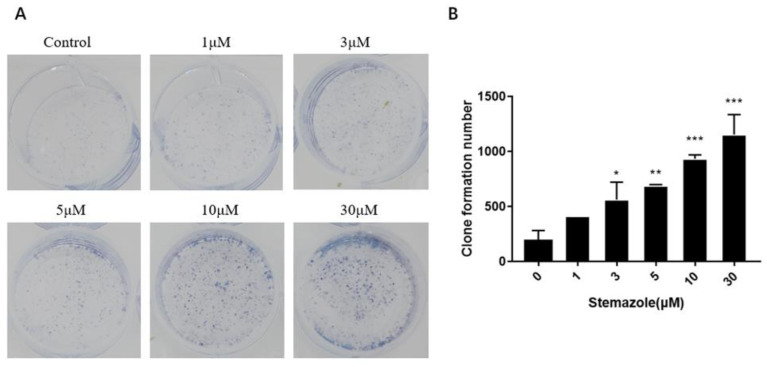
Stemazole promotes colony formation in OPCs. (**A**) Colony formation in OPCs as assessed by Giemsa staining. (**B**) Dose-dependent effects on the clone formation of monoclonal colonies. Data represent the means ± SD (*n* = 3 duplicates), * *p* < 0.05, ** *p* < 0.01, *** *p* < 0.001. vs. 0 μM group.

Colony formation experiments reflect the survival rate of cell inoculation, indicating the number of adherent cells that survive and form clones after inoculation. To further investigate the effect of stemazole on OPCs, we inoculated disaggregated OPCs in a medium with different concentrations of stemazole for 4 days. The Giemsa staining results showed that stemazole increased the number of OPCs in a dose-dependent manner (Figure 2A,B). In contrast, most OPCs did not survive in disaggregated cell cultures without stemazole. Specifically, compared with cells cultured without stemazole, the colony formation rate was considerably increased after treatment with stemazole (1, 3, 5, 10, and 30 μM), achieving approximately 226%, 375%, 503%, 746%, and 966% improvement, respectively. A significant increase in OPCs was observed when the dose of stemazole was 3 μM (*p* < 0.05). Moreover, the colony formation ability was increased by approximately 6-fold upon treatment with 30 μM stemazole compared with untreated OPCs (*p* < 0.001). Taken together, when cultured with stemazole, the individual OPCs were more likely to be adherent and clonal. These results indicate that stemazole notably promotes the survival of disaggregated OPCs in a dose-dependent manner.

### 2.3. Stemazole Prevents Apoptosis of Oligodendrocyte Precursor Cells

**Figure 3:**
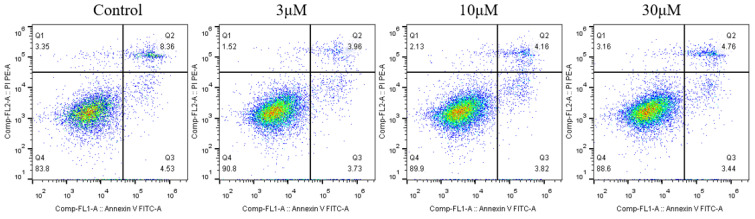
OPCs were treated with stemazole for 4 days, and apoptosis was analyzed by flow cytometry after staining with annexin V and propidium iodide. Values shown are the percentage of cells stained with annexin V.

Apoptotic cells were analyzed by flow cytometry after annexin V and propidium iodide staining. As shown in Figure 3, the apoptosis distribution results show that the percentages of early and late apoptosis were 12.89% in the control group, 7.69% in the 3 μM stemazole group, 7.98% in the 10 μM stemazole group and 8.2% in the 30 μM stemazole group. Different concentrations of stemazole (3, 10, and 30 μM) reduced the proportion of early and late apoptotic cells. Therefore, 3 μM stemazole decreased the percentage of cell apoptosis by 5.2% and served as the optimal concentration. The above results suggest that the proportion of apoptotic cells reached 12.89% in the culture condition of 10% serum, which caused slight damage to the cells. The addition of the small molecule stemazole conferred resistance to apoptosis in disaggregated OPCs.

### 2.4. Stemazole Improves Motor Dysfunction and Neurological Impairment In Vivo

Given that MS patients have the pathological characteristics of demyelination and the clinical symptoms of paralysis and cognitive neurological disorders, we chose the CPZ model to simulate the clinical symptoms and demyelination process of the patients. Moreover, to investigate the therapeutic effect of stemazole on the demyelination model, we studied the recovery of motor coordination and memory impairment in behavioral tests and remyelination degree in histological tests on CPZ-mediated demyelination animals.

### 2.5. Stemazole Improves Motor Coordination

**Figure 4:**
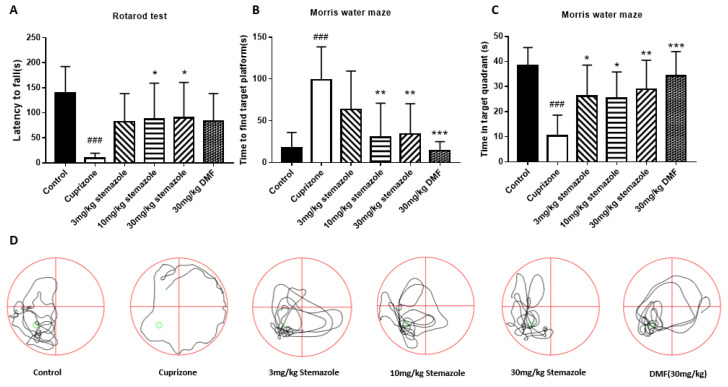
Function of stemazole on CPZ-induced spatial cognition and memory impairment by the Morris water maze task and rotarod test. Latency to fall (**A**), Time to find the target platform (**B**), Number of platform crossings (**C**), and Swimming tracks (**D**) were recorded via a video tracking camera system. Data represent the means ± SD (*n* = 10 duplicates). * *p* < 0.05, ** *p* < 0.01, *** *p* < 0.001 vs. the CPZ group. ^###^ *p* < 0.001 vs. the control group.

The rotarod test was used to determine the movement coordination ability of the experimental animals. Compared with the control group, a significant decrease in the CPZ group was noted over time, achieving an approximately 130 s decline (*p* < 0.001). Compared with the CPZ group, the mice treated with stemazole at doses of 3, 10 and 30 mg/kg had longer persistence times, achieving approximately 72.63 s, 77.25 s and 80.13 s improvements, respectively. Compared with the CPZ group, the endurance time of the DMF group increased by 74.13 s, however, this result was not statistically significant (Figure 4A). After the induction of CPZ, the mice in the CPZ model group could hardly persist on the rotarod. Compared with the CPZ group, either 10 mg/kg or 30 mg/kg stemazole significantly promoted motor coordination and balance ability. However, 30 mg/kg DMF did not enhance the movement coordination ability. These results show that stemazole significantly recovered the movement coordination ability of demyelination animals in the CPZ model, which had a better effect than the positive drug.

### 2.6. Stemazole Improves Memory Impairment

The Morris water maze (MWM) was used to test the learning and memory abilities of the experimental animals. To measure the learning and memory ability of mice, the time that the mice spent finding the platform was recorded. According to the results, the mice treated with 10 and 30 mg/kg stemazole exhibited a significantly shorter time to find the platform than the CPZ group, with reductions of 68.25 s and 64.75 s, respectively. Mice in the DMF group had the best recovery effect, reaching a healthy level by extending the time of searching for the platform by 84.63 s. Compared with the CPZ group, the stemazole and DMF treatments both reduced the time required by mice to find the platform (Figure 4B).

To evaluate the ability of the mice to retain their memory of the platform’s spatial location after learning to find it, the platform was removed. Then, the paths the mice swam were observed (Figure 4D). The CPZ group circled the edge, and the other five groups repeatedly passed by the platform. Compared with the control group, the stay time of the mice in the CPZ group was significantly reduced by 25% (Figure 4C). Treatment with 3, 10 and 30 mg/kg stemazole enhanced the stay time by 15.73 s, 14.8 s and 18.52 s, respectively. The stay time of the DMF group increased up to 23.85 s (*p* < 0.001). The mice in the CPZ group had the least stay time in the target quadrant. The mice in the control group, stemazole group and DMF group had a longer stay time in the target quadrant.

The aforementioned statistics demonstrate that CPZ intoxication reduced the animal capacity of spatial learning, memory and spatial impairment. After treatment with stemazole and DMF, the above symptoms were significantly ameliorated.

### 2.7. Stemazole Promotes Remyelination

The most typical pathological histological feature of MS is myelin loss. The assessments of myelin, MBP proteins and OL-specific transcription factor (Olig2) [25,26] are key indicators for the identification of remyelination. Therefore, we used histology, immunochemistry and immunofluorescence staining to comprehensively evaluate remyelination.

**Figure 5:**
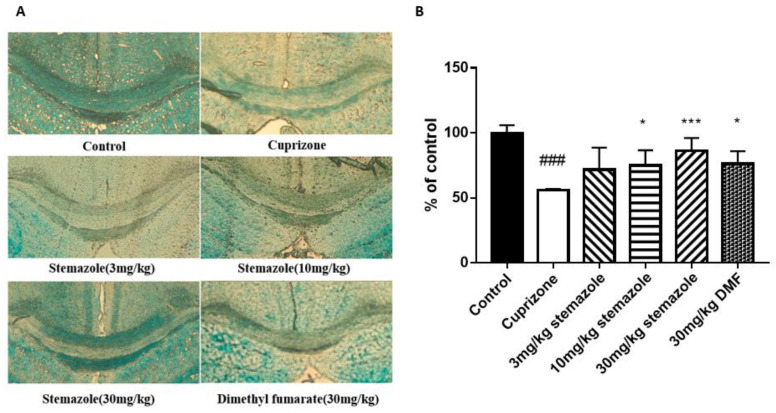
The protective effect of stemazole on myelin in corpus callosum (cc) demyelinating lesions was observed by LFB staining. (**A**) LFB staining was employed to evaluate the severity of demyelination. (**B**) Myelin densities were compared with those of the control and expressed as a percentage of the control value using ImageJ software. Data represent the means ± SD (*n* = 5 duplicates). * *p* < 0.05, *** *p* < 0.001 vs. the CPZ group. ^###^ *p* < 0.001 vs. control group.

We investigated the myelin status in the corpus callosum region using Luxol Fast Blue staining. The data indicated a significant loss of myelin in the CPZ group, with a 56.02% decrease compared with the control group. Compared with the CPZ group, the myelin area of the mice treated with 10 and 30 mg/kg stemazole was increased by 19.85% and 30.46%, respectively. Compared with the CPZ group, the myelin area of the mice treated with DMF was increased by 20.22%. Above all, compared with the control group, approximately 50% damage was induced by CPZ. After CPZ removal, stemazole substantially accelerated the remyelination process in a dose-dependent manner (Figure 5). Stemazole (30 mg/kg) significantly promoted remyelination in the corpus callosum, which had better effects than the same dose of the positive control drug.

**Figure 6:**
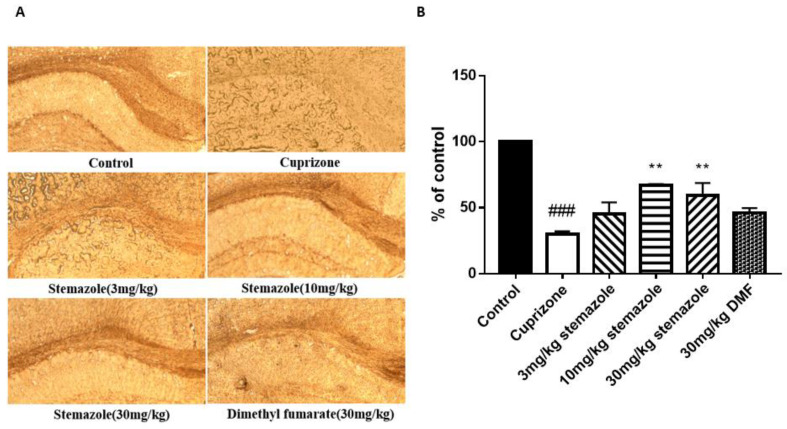
The protective effect of stemazole on myelin from demyelinated lesions in the corpus callosum (cc) by MBP immunohistochemistry. (**A**) MBP immunohistochemistry was used to evaluate the severity of demyelination. (**B**) MBP expression levels were compared with those of controls and expressed as a percentage of the control value using the ImageJ analysis program. Data represent the means ± SD (*n* = 3 duplicates). ** *p* < 0.01 vs. the CPZ group. ^###^ *p* < 0.001 vs. control group.

We further studied the degree of remyelination in the corpus callosum after 2 weeks of stemazole treatment using the immunohistochemical staining of the myelin marker MBP (Figure 6). The data indicate a 29.9% increase in MBP expression in the CPZ group compared with the control group; treatment with 3, 10 and 30 mg/kg stemazole promoted remyelination with improvements of 15.45%, 37.08% and 29.35%, respectively, in comparison with the CPZ group. The area of myelin in the DMF group increased the expression of MBP up to 16.28%. The data indicate that CPZ could decrease the expression of MBP up to 70.1% compared with the healthy level. Treatment with 10 mg/kg and 30 mg/kg stemazole clearly promoted MBP expression. Compared with 30 mg/kg stemazole, the same dose of the positive control drug did not show a significant effect on promoting myelin regeneration. These results show that stemazole could significantly promote myelin regeneration and had a better effect than the positive control drug.

**Figure 7:**
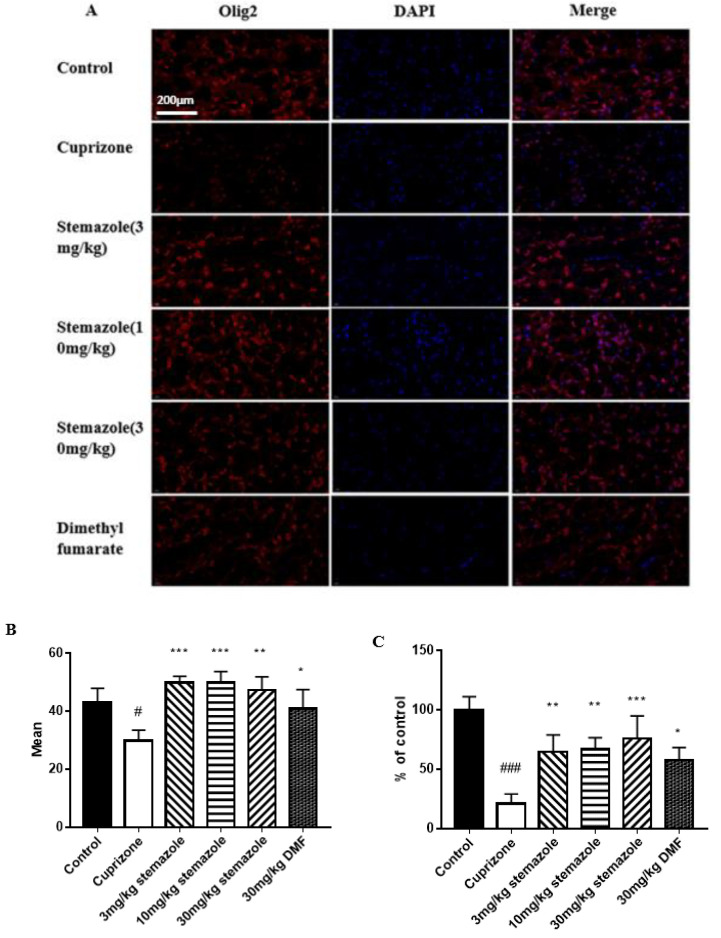
The promoting effect of stemazole on the expression of the OL-specific transcription factor Olig2 by immunofluorescence. (**A**) Olig2 immunofluorescence to assess Olig2 expression. (**B**) The average fluorescence intensity of Olig2 expression. (**C**) The olig2 cells/mm^2^ were compared with that of the control and expressed as a percentage of the control value using ImageJ software. Data represent the means ± SD (*n* = 3 duplicates). * *p* < 0.05, ** *p* < 0.01 *** *p* < 0.001 vs. the CPZ group. ^#^ *p* < 0.05, ^###^ *p* < 0.001 vs. control group.

We also evaluated the Olig2 expression using immunofluorescence staining (Figure 7). The results indicate a significant reduction in Olig2 expression in the CPZ group compared to the control group (42.94 vs. 29.91, *p* < 0.05). The group treated with stemazole (3, 10 and 30 mg/kg) showed an improvement in Olig2 expression, achieving approximately 1.66-, 1.66- and 1.57-fold increases, respectively. A significant increase in the DMF group (1.33 times, *p* < 0.05) was also observed. In addition, the group treated with stemazole (3, 10 and 30 mg/kg) showed an improvement in Olig2 cells/mm^2^, achieving approximately 3.09-, 3.19- and 3.64-fold increases. The results show that treatment with stemazole increased the expression of oligodendrocytes (OLs) more effectively than the positive control drug.

Taken together, stemazole recovered the learning ability and movement coordination ability of demyelination animals. In addition, stemazole increased the expression of OLs and promoted remyelination by histology. Moreover, stemazole performed significantly better than positive control drugs in promoting the recovery of myelin.

## 3. Discussion

Stemazole is a novel small molecule previously found to have neuroprotective effects against neurodegeneration [20]. In our research, we studied the effect of stemazole on promoting OPC survival in vitro and remyelination in vivo to investigate its therapeutic potential for multiple sclerosis. The results show that stemazole promotes the survival, attachment and apoptosis resistance of OPCs. Stemazole enhanced the cell viability, formation number, and colony size in a dose-dependent manner. In addition, 10 and 30 mg/kg stemazole promoted the recovery of motor dysfunction and remyelination in the CPZ-induced demyelination model, which performed better than DMF. This finding indicates that stemazole promotes oligodendrocyte precursor cell survival, protects the cells from damage in vitro and improves motor dysfunction and neurological impairment in vivo.

Neuroprotection and myelin regeneration are therapeutic strategies that are widely studied in the treatment of multiple sclerosis. OPCs undergo cell death upon disaggregation into single cells or upon starvation, precluding differentiation. According to the in vitro results, OPCs caused slight injury and apoptosis in 10% serum culture. Stemazole significantly enhanced cell viability, increased the number of colonies formed in a dose-dependent manner and antagonized the proportion of apoptotic cells. In our previous study, the results indicated that stemazole could antagonize cell apoptosis by reducing the caspase 2 protein, which is well known to be involved in apoptosis [27]. Stemazole could also prevent cell apoptosis due to disaggregation or starvation without compromising self-renewal, proliferation and pluripotency [23]. For this reason, we believe that stemazole can promote cell survival and maintain the normal function of cells.

CPZ is a copper chelating agent commonly used as a neurotoxicant to induce demyelination and neurodegeneration [28]. The CPZ model simulates the process of demyelination and remyelination and serves as a suitable model for neuroprotection and myelin regeneration therapy. In the past 20 years, CPZ model studies have been widely conducted to develop neuroprotective therapies. In this study, we selected a CPZ-induced model to study whether stemazole can promote remyelination and restore nerve function. According to the in vitro results, stemazole significantly prolonged the persistence time on the rotarod, reduced the time to find the platform and increased the time in the quadrant of the original platform in behavioral studies, suggesting that stemazole effectively improved animal motor balance and the learning and memory ability of demyelinated mice. Histologically, stemazole promoted myelin area, MBP content and Olig2 expression in demyelinated mice. Compared with the CPZ group, stemazole increased the myelin area by 30.46% (*p* < 0.001), MBP expression by 37.08% (*p* < 0.01) and Olig2 expression by 1.6-fold (*p* < 0.001). However, compared with the stemazole group, the DMF group did not show a better promoting effect on myelin area, Olig2 expression or MBP content. Stemazole both promoted OPC survival in vitro and protected myelin from CPZ-induced toxicity in vivo. Stemazole has revealed pharmacological activity in preventing and treating neurodegenerative diseases by promoting the repair potential of cells. The neuroprotective effects of stemazole may be important for its therapeutic efficacy.

DMF is an approved drug that relieves neuroinflammation and oxidative stress damage to reduce nerve injury [15]. Because DMF has neuroprotective pharmacological activity, the drug was selected as a positive control. According to the behavioral studies, DMF almost restored neurological function to a healthy level. According to histological studies, DMF could partially reverse the damage to the myelin sheath. The recovery effect of DMF is still unable to reach a healthy level and is slightly inferior to the effect of stemazole. We hypothesize that DMF could restore neurological function, which may be related to its ability to alleviate neuroinflammation and antioxidative damage caused by CPZ. However, DMF does not promote the regeneration of myelin; thus, it cannot restore myelin to a healthy level. Although DMT drugs, such as DMF, modulate the immune process to alleviate disease recurrence, the effect of promoting myelin regeneration is not sufficient. Thus, more work on drug screening is urgently needed.

A limitation of this study was that it only studied the pharmacodynamics of stemazole, and its mode of action needs to be explored.

## 4. Materials and Methods

### 4.1. Chemicals and Reagents

Stemazole was synthesized by the Key Laboratory of Radiopharmaceuticals. The purity of stemazole was determined to be >98% by high-performance liquid chromatography (HPLC), and the structure of stemazole was confirmed by high-resolution mass spectrometry and nuclear magnetic resonance. CPZ, Giemsa, and Luxol Fast Blue were purchased from Sigma–Aldrich (St. Louis, MO, USA). The rabbit anti-MBP antibody (Cat No. 10458-1-AP), rabbit anti-Olig2 antibody (Cat No. 13999-1-AP), immunohistochemistry kit and immunofluorescence kit were purchased from Proteintech (Wuhan, China).

### 4.2. Cell Assay

#### 4.2.1. OPC Culture

OPCs were purchased from Otwobiotech (Shenzhen, China). The purity of this cell line was determined to be >90%. The cells were maintained in 90% DMEM-HG supplemented with 10% FBS at 37 °C in a 5% CO_2_-humidified atmosphere.

#### 4.2.2. Cell Viability Assay

The cells were seeded at 3000 cells per well in a flat-bottomed 96-well plate and exposed to 1, 3, 5, 10, 15, 20, 25 or 30 μM stemazole dissolved in PBS for 4 days. Cell viability was quantitatively analyzed using the Cell Titer-Glo™ luminescent cell viability assay, which is based on the luciferase/luciferin reaction. After mixing and incubating for 10 min at ambient temperature, the luminescence value was measured at 595 nm with a Multilabel Counter (PE, 1420, VICTOR3™).

#### 4.2.3. Clone Formation Assay

To assess clone formation, 1000 single cells were seeded into a flat-bottomed 6-well plate and exposed to 1, 3, 5, 10 or 30 μM stemazole for 4 days. The day on which OPCs were seeded was defined as Day 0. The number of colonies formed was measured by Giemsa staining.

#### 4.2.4. Annexin V-PE Apoptosis Detection Assay

For annexin V staining and fluorescence-activated cell sorting, dissociated cells were seeded at 3 × 10^5^ cells in a 60 mm dish and treated with 3, 10, or 30 μM stemazole for 4 days. The cells were then collected, resuspended, centrifuged for 5 min, the supernatant was discarded, and the cells were gently resuspended by adding 195 μL Annexin V-PE binding solution. Then, 5 μL Annexin V-PE was added. The samples were incubated for 10–20 min at room temperature in the dark. Flow cytometry was performed immediately, and Annexin V-PE showed red fluorescence. The day on which OPCs were seeded was defined as Day 0.

### 4.3. Animals and Treatment Procedures

**Figure 8:**
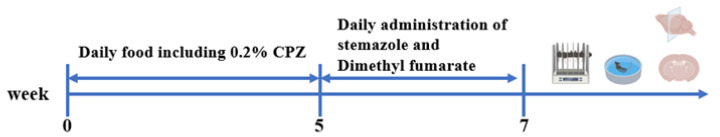
A diagram of the cuprizone-induced demyelination model and the administration of stemazole.

Female C57BL/6 mice (8 weeks) were fed 0.2% (*w/w*) cuprizone mixed with ground standard rodent chow. The CPZ diet was maintained for 5 weeks, after which the CPZ-infused feed was removed, and the animals were given standard chow. The mice were administered a daily intraperitoneal injection of stemazole dissolved in normal saline (3, 10 and 30 mg/kg) and a daily intragastric injection of DMF (30 mg/kg) after CPZ withdrawal. The treatment lasted for 2 weeks. Experimental procedures involving animals were approved by the Ethics Committee of Beijing Normal University (BNU/EC/01/2011) (Figure 8).

### 4.4. Behavioral Test

#### 4.4.1. Rotarod Test

Motor function was assessed using the rotarod test. Before the formal experiment, the mice (*n* = 10) were trained to adapt to the rotating cylinder environment operating at 20 rpm for 3 consecutive days by DXP-3 (Chinese Academy of Medical Sciences, Beijing, China). The movement was maintained for 300 s. During the formal test periods, the time spent on the rotating bar until the mice fell from the cylinder was recorded.

#### 4.4.2. Morris Water Maze

Spatial learning and memory were tested using the Morris water maze behavior test. The water maze device (Chinese Academy of Medical Sciences, China) consists of a circular pool divided into four quadrants, and a translucent platform below the water surface was hidden in the center of the quadrant. In the navigation task, the time required for the mice to find the underwater platform within a 120 s period was recorded. In the exploration experiment, the platform was removed, and the time spent by the animal in the target quadrant (the quadrant where the platform was originally placed) and the number of times it entered the quadrant within 60 s were recorded.

### 4.5. Histology, Immunochemistry and Immunofluorescence Staining

#### 4.5.1. Luxol Fast Blue (LFB) Staining

LFB was performed to evaluate the degree of demyelination. The sections (*n* = 5) were immersed in 0.01% LFB solution and incubated at 60 °C for 2 h. The sections were differentiated in lithium carbonate solution for 20 s followed by 70% ethyl alcohol for 20 s. The sections were dehydrated in graded alcohols. Then, the samples were dehydrated, cleared and mounted.

#### 4.5.2. Immunochemistry Staining

Immunochemistry staining was performed to evaluate MBP expression. The sections (*n* = 3) were fixed in 4% paraformaldehyde, incubated with rabbit anti-MBP (1:200) antibody at 4 °C overnight and stained with an immunohistochemistry kit following the manufacturers’ instructions.

#### 4.5.3. Immunofluorescence Staining

Immunofluorescence staining was performed to evaluate the expression of the transcription factor Olig2. The sections (*n* = 3) were fixed in 4% paraformaldehyde, incubated with rabbit anti-Olig2 (1:200) antibody at 4 °C overnight and stained with an immunofluorescence kit following the manufacturers’ instructions.

### 4.6. Data Analysis

In our experiments, the data were analyzed using GraphPad Prism 5. Data are presented as the means ± SD. Differences between groups were analyzed using one-way ANOVA followed by Dunnett’s post hoc test. Student’s *t* test was used to compare two sets of data. A value of *p* < 0.05 was regarded as significant.

## 5. Conclusions

In conclusion, our experimental data show that stemazole promotes OPC survival in vitro, improves behavioral motor dysfunction and reduces the degree of demyelination in vivo. These results indicate that stemazole may be used as a potential therapeutic agent against MS.

## Data Availability

The data that support the findings of this study are available from the corresponding author upon reasonable request.

## References

[1] Zoupi L., Booker S.A., Eigel D., Werner C., Kind P.C., Spires-Jones T.L., Newland B., Williams A.C. (2021). Selective vulnerability of inhibitory networks in multiple sclerosis. Acta Neuropathol..

[2] Thompson A.J., Baranzini S.E., Geurts J., Hemmer B., Ciccarelli O. (2018). Multiple sclerosis. Lancet.

[3] Walton C., King R., Rechtman L., Kaye W., Leray E., Marrie R.A., Robertson N., La Rocca N., Uitdehaag B., van der Mei I. (2020). Rising prevalence of multiple sclerosis worldwide: Insights from the Atlas of MS, third edition. Mult. Scler. J..

[4] Franklin R.J.M., ffrench-Constant C. (2008). Remyelination in the CNS: From biology to therapy. Nat. Rev. Neurosci..

[5] Lassmann H. (2019). Pathogenic Mechanisms Associated with Different Clinical Courses of Multiple Sclerosis. Front. Immunol..

[6] Nave K.-A. (2010). Myelination and the trophic support of long axons. Nat. Rev. Neurosci..

[7] Saab A.S., Tzvetanova I.D., Nave K. (2013). The role of myelin and oligodendrocytes in axonal energy metabolism. Curr. Opin. Neurobiol..

[8] Rosenberg S.S., Kelland E.E., Tokar E., De La Torret A.R., Chan J.R. (2008). The geometric and spatial constraints of the microenvironment induce oligodendrocyte differentiation. Proc. Natl. Acad. Sci. USA.

[9] Ferent J., Zimmer C., Durbec P., Ruat M., Traiffort E. (2013). Sonic Hedgehog Signaling Is a Positive Oligodendrocyte Regulator during Demyelination. J. Neurosci..

[10] Skaper S.D. (2019). Oligodendrocyte precursor cells as a therapeutic target for demyelinating diseases. Prog. Brain Res..

[11] Elbaz B., Popko B. (2019). Molecular Control of Oligodendrocyte Development. Trends Neurosci..

[12] Faissner S., Plemel J.R., Gold R., Yong V.W. (2019). Progressive multiple sclerosis: From pathophysiology to therapeutic strategies. Nat. Rev. Drug Discov..

[13] Scannevin R.H., Chollate S., Jung M., Shackett M., Patel H., Bista P., Zeng W., Ryan S., Yamamoto M., Lukashev M. (2012). Fumarates Promote Cytoprotection of Central Nervous System Cells against Oxidative Stress via the Nuclear Factor (Erythroid-Derived 2)-Like 2 Pathway. J. Pharmacol. Exp. Ther..

[14] Derfuss T., Mehling M., Papadopoulou A., Bar-Or A., Cohen J.A., Kappos L. (2020). Advances in oral immunomodulating therapies in relapsing multiple sclerosis. Lancet Neurol..

[15] Schulze-Topphoff U., Varrin-Doyer M., Pekarek K., Spencer C.M., Shetty A., Sagan S.A., Cree B.A.C., Sobel R.A., Wipke B.T., Steinman L. (2016). Dimethyl fumarate treatment induces adaptive and innate immune modulation independent of Nrf2. Proc. Natl. Acad. Sci. USA.

[16] Chen Y., Kuchroo V., Inobe J., Hafler D., Weiner H. (1994). Regulatory t-cell clones induced by oral tolerance: Suppression of autoimmune encephalomyelitis. Science.

[17] Benetti F., Ventura M., Salmini B., Ceola S., Carbonera D., Mammi S., Zitolo A., D’Angelo P., Urso E., Maffia M. (2010). Cuprizone neurotoxicity, copper deficiency and neurodegeneration. Neurotoxicology.

[18] Koutsoudaki P.N., Skripuletz T., Gudi V., Moharregh-Khiabani D., Hildebrandt H., Trebst C., Stangel M. (2009). Demyelination of the hippocampus is prominent in the cuprizone model. Neurosci. Lett..

[19] Zhang J., Li H., Zhang Y., Zhao C., Zhu Y., Han M. (2020). Uncovering the Pharmacological Mechanism of Stemazole in the Treatment of Neurodegenerative Diseases Based on a Network Pharmacology Approach. Int. J. Mol. Sci..

[20] Han M., Liu Y., Tan Q., Zhang B., Wang W., Liu J., Zhang X., Wang Y., Zhang J. (2011). Therapeutic efficacy of stemazole in a beta-amyloid injection rat model of Alzheimer’s disease. Eur. J. Pharmacol..

[21] Guo Z., Xu S., Du N., Liu J., Huang Y., Han M. (2016). Neuroprotective effects of stemazole in the MPTP-induced acute model of Parkinson’s disease: Involvement of the dopamine system. Neurosci. Lett..

[22] Li H., Tan Q., Zhang Y., Zhang J., Zhao C., Lu S., Qiao J., Han M. (2020). Pharmacokinetics and absolute oral bioavailability of stemazole by UPLC-MS/MS and its bio-distribution through tritium labeling. Drug Test. Anal..

[23] Sun Y., Zhang X., Li H., Xu S., Zhang X., Liu Y., Han M., Wen J. (2018). Stemazole promotes survival and preserves stemness in human embryonic stem cells. FEBS J..

[24] Sun Y., Wang W., Sun Y., Han M. (2011). Synthesis and biological evaluation of a novel human stem/progenitor cells proliferation activator: 4-(4-(5-mercapto-1,3,4-oxadiazol-2-yl)phenyl) thiosemicarbazide (Stemazole). Eur. J. Med. Chem..

[25] Wegener A., Deboux C., Bachelin C., Frah M., Kerninon C., Seilhean D., Weider M., Wegner M., Nait Oumesmar B. (2015). Gain of Olig2 function in oligodendrocyte progenitors promotes remyelination. Brain.

[26] Zhu X., Zuo H., Maher B.J., Serwanski D.R., LoTurco J.J., Lu Q.R., Nishiyama A. (2012). Olig2-dependent developmental fate switch of NG2 cells. Development.

[27] Chen M., Zhu Y., Li H., Zhang Y., Han M. (2022). A Quantitative Proteomic Approach Explores the Possible Mechanisms by Which the Small Molecule Stemazole Promotes the Survival of Human Neural Stem Cells. Brain Sci..

[28] Zendedel A., Beyer C., Kipp M. (2013). Cuprizone-Induced Demyelination as a Tool to Study Remyelination and Axonal Protection. J. Mol. Neurosci..

